# Glissades Are Altered by Lesions to the Oculomotor Vermis but Not by Saccadic Adaptation

**DOI:** 10.3389/fnbeh.2019.00194

**Published:** 2019-08-23

**Authors:** Nico A. Flierman, Alla Ignashchenkova, Mario Negrello, Peter Thier, Chris I. De Zeeuw, Aleksandra Badura

**Affiliations:** ^1^Department of Neuroscience, Erasmus MC, Rotterdam, Netherlands; ^2^Netherlands Institute for Neuroscience, Amsterdam, Netherlands; ^3^Department of Cognitive Neurology, Hertie Institute for Clinical Brain Research, Tübingen, Germany; ^4^Werner Reichardt Centre for Integrative Neuroscience, University of Tübingen, Tübingen, Germany

**Keywords:** cerebellum, lesion, vermis, saccades, glissade, adaptation, motor learning

## Abstract

Saccadic eye movements enable fast and precise scanning of the visual field, which is partially controlled by the posterior cerebellar vermis. Textbook saccades have a straight trajectory and a unimodal velocity profile, and hence have well-defined epochs of start and end. However, in practice only a fraction of saccades matches this description. One way in which a saccade can deviate from its trajectory is the presence of an overshoot or undershoot at the end of a saccadic eye movement just before fixation. This additional movement, known as a glissade, is regarded as a motor command error and was characterized decades ago but was almost never studied. Using rhesus macaques, we investigated the properties of glissades and changes to glissade kinematics following cerebellar lesions. Additionally, in monkeys with an intact cerebellum, we investigated whether the glissade amplitude can be modulated using multiple adaptation paradigms. Our results show that saccade kinematics are altered by the presence of a glissade, and that glissades do not appear to have any adaptive function as they do not bring the eye closer to the target. Quantification of these results establishes a detailed description of glissades. Further, we show that lesions to the posterior cerebellum have a deleterious effect on both saccade and glissade properties, which recovers over time. Finally, the saccadic adaptation experiments reveal that glissades cannot be modulated by this training paradigm. Together our work offers a functional study of glissades and provides new insight into the cerebellar involvement in this type of motor error.

## Introduction

**FIGURE 1:**
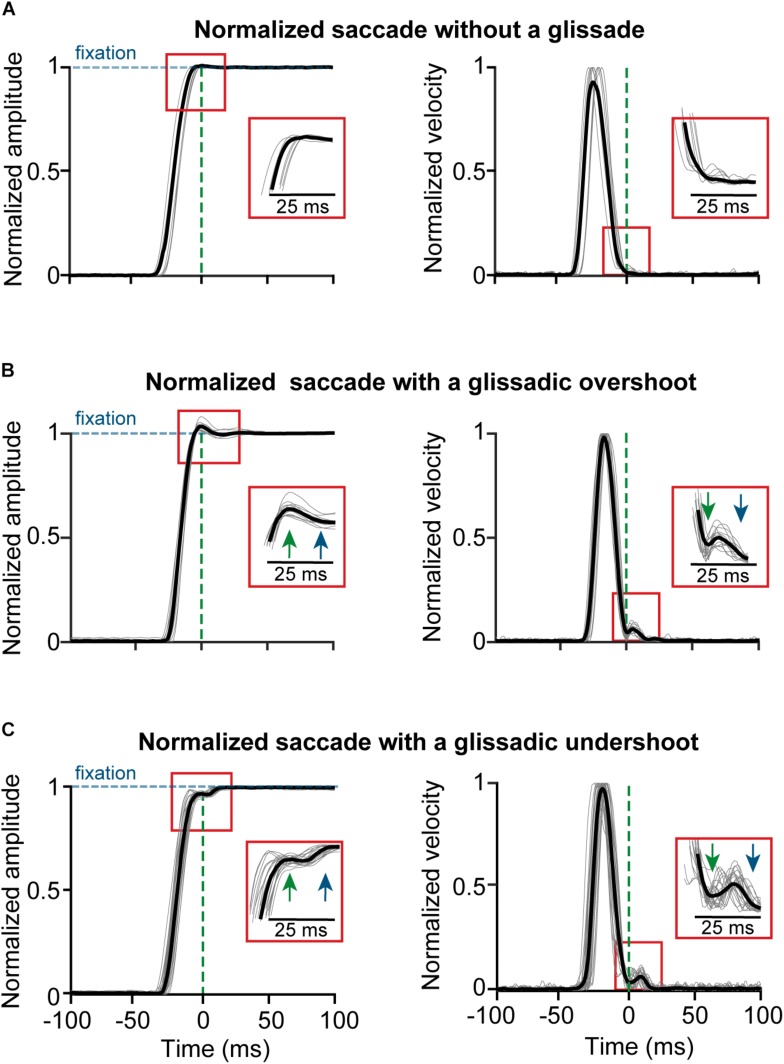
Example traces of saccades normalized to fixation with and without the glissade. All saccades aligned to the onset of a glissade. **(A)** Position and velocity traces of saccade (gray) and average (black) of saccades without a glissade; 59% of all saccades. **(B)** Example traces of saccades with glissadic overshoot (35% of all saccades); inset shows glissade onset (green arrow) and offset (blue arrow). **(C)** Example traces of saccades with a glissadic undershoot (6% of all saccades). Percentages were calculated from 3597 saccades from 4 monkeys (data collected with a scleral search coil). Traces were normalized to prevent the glissades from being concealed by the variability in saccade amplitude.

High-resolution vision is limited to the foveal region of the retina. Therefore, the visual system depends on saccadic eye movements to scan regions of interest in the visual field with high resolution. Since visual input is unavailable during saccades (Volkmann, 1962) and it is ethologically relevant to spend as little time blind as possible, gaze shifts need to have high velocity and accuracy. Saccades are some of the fastest movements that a body can produce, with durations shorter than 60 ms (Robinson, 1964), and amplitudes ranging from tenths of a degree (microsaccades) up to 60 degrees (Bahill et al., 1975c). The end-points of these ultra-fast movements regularly contain slow drifting overshoots or undershoots relative to fixation, which are referred to as glissades (Figure 1) (Weber and Daroff, 1972; Bahill et al., 1975b; Hsu and Stark, 1978).

From a historic perspective the term glissade was first used by Weber and Daroff (1972) analogizing the long and slow post-saccadic drifts with a ‘*glissando’* on the piano, where the fingers glide from one note to another. The shorter duration, zero-latency “dynamic over- and undershoot,” was initially referred to as a separate phenomenon. Indeed, Bahill and colleagues’ came up with two distinct models for each of these descriptions (Bahill and Stark, 1975; Bahill et al., 1975a). Later work often omitted the distinction due to empirical considerations, simply referring to both as a “glissade” (Kapoula et al., 1986; Nyström and Holmqvist, 2010). The main reason for the lack of distinction is that even if they are functionally distinguishable phenomena, the heterogeneity of the eye motion kinematics makes a meaningful separation virtually impossible in eye tracking data.

Here, we consider a glissade a drift-like movement that immediately follows the end of the decelerating phase of the saccade and before the eye settles on the final point (see section Materials and Methods for details on the detection procedure and criteria). Figure 1 shows examples of saccades without glissades (top panels) as well as saccades with glissadic overshoots (middle panels) and undershoots (middle and bottom panels; all saccades are aligned to the glissade onset) from the dataset used in this paper. Glissade duration, amplitudes and peak velocities occur in the same range as those of micro-saccades (Tian et al., 2018).

Glissades have marked importance in the context of precision, programing, and the relationship of saccades to other eye movements. The study of glissades is timely: delineating the start and end of the saccade is an issue that repeatedly comes up in the recent surge of eye movement trackers with head-free and even freely moving subjects (Chukoskie et al., 2018; Macinnes et al., 2018; Wang et al., 2019) (for a review of commercially available eye tracking software used in research and commercial applications see^1^). This is particularly relevant for studies where eye movements are proposed to be used as a diagnostic criteria (Klin et al., 2009; Al-Wabil and Al-Sheaha, 2010). In these conditions stationary fixation preceding and following a saccade is the exception. More commonly the eye moves both before and after the saccade. These movements often comprise compensatory eye movement and other factors. Since modern technology and research makes frequent use of eye-tracking systems, segregating saccades becomes an important problem, which is anything but straightforward. In this context, recognizing glissades and understanding their basic properties and relationship to saccades is of high importance.

So far the only physiological investigation of glissades describes the role of the lateral intraparietal cortex, an area that is known for visual saliency maps and attention, but also participates in the planning of saccades (O’Leary and Lisberger, 2012). The eye movements they study are in the range of 2–4°/s, whereas the eye movements in our study and those reported in the literature are around 20°/s (Nyström and Holmqvist, 2010). It is therefore possible that the 2012 study by O’Leary and Lisberger focuses more on the slow and long post-saccading drifts (Weber and Daroff, 1972), ignoring the zero-latency “dynamic over- and undershoots” in their definition of the glissade. This discrepancy makes the argument for unifying the two definitions even more pressing.

**FIGURE 2:**
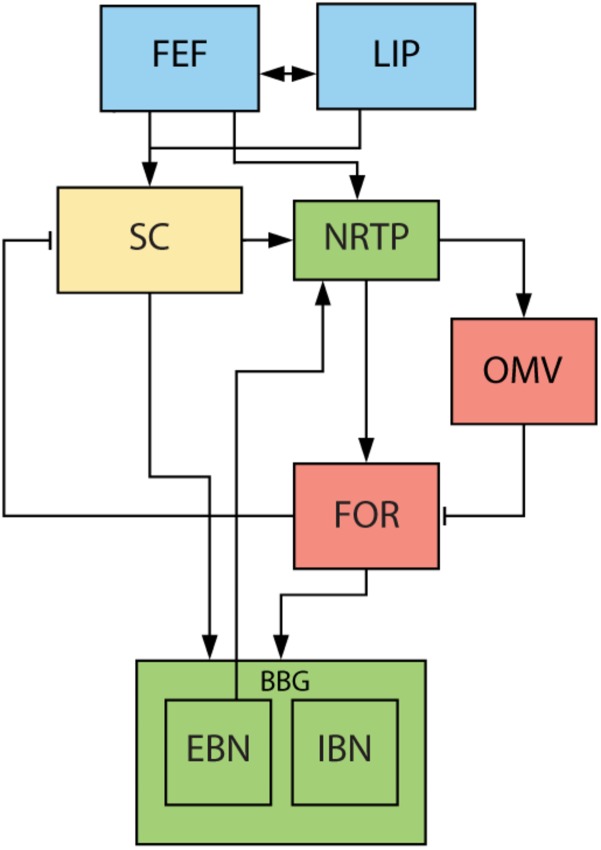
Overview of saccade related areas in the midbrain, brainstem, and cerebellum. Saccade targets are jointly selected by cerebral cortex (blue) and SC (yellow). Subsequently, SC, brainstem (green) and cerebellum (red) execute the movement. SC provides drive to premotor nuclei and cerebellum via NRTP. Cerebellum provides additional drive to premotor nuclei through the FOR. EBN give feedback signal back to cerebellum about the progress of the saccade. FEF, frontal eye fields. LIP, lateral intraparietal cortex. SC, superior colliculus. NRTP, nucleus reticularis tegmenti pontis. OMV, oculomotor vermis. FOR, fastigial oculomotor region. EBN, excitatory burst neurons. IBN, inhibitory burst neurons. Blue areas are in the cerebral cortex, yellow areas represent the midbrain, green areas the brainstem, and red areas part of the cerebellum.

There is little known about the role of downstream structures, responsible for the execution of a saccade, in the generation of glissades. One of the major hubs in this complex network for planning and execution of saccades is the cerebellum (Figure 2). It is responsible for the fine-tuning of oculomotor performance and for keeping saccades accurate despite changes in the oculomotor system due to development, aging or disease (Golla et al., 2008; Beh et al., 2017). Lesions to the cerebellum have been shown to decrease saccadic accuracy and affect the amplitude and kinematic properties of saccades. Additionally, the cerebellum is also responsible for the adaptive lengthening and shortening of saccades based on a visual error, also known as saccadic adaptation (Pélisson et al., 2010). The posterior part of cerebellar vermis (lobules VIc, VII), also known as the oculomotor vermis (OMV), is a part of the cerebellum that is responsible for the control of saccadic eye movements. Lesions to this part of the cerebellum cause transient dysmetria (hypo- and/or hypermetria) and abolish the capacity for adaptive lengthening, but not shortening, of saccadic amplitudes through saccadic adaptation (Barash et al., 1999; Ignashchenkova et al., 2009).

The motoneuronal control of saccades has been studied in detail and is much better understood. It is commonly understood as consisting of two components, a phasic ‘pulse’ and a tonic ‘step’ (for a schematic overview of brain regions involved see Figure 2). The pulse brings the eyes to the new position and is characterized by a high frequency burst in the motoneurons (Sindermann et al., 1978; Scudder et al., 2002). The duration of this burst is approximately equal to the duration of the saccade, and it is accompanied by inhibition of the motoneuronal activity for the antagonist muscle. The step is a tonic activation which holds the eye in its new position (Van Gisbergen et al., 1981; Sparks, 2002). If the two components are matched, the saccade lands exactly on target. However, if the pulse is stronger than the step, the eye travels beyond its intended position and slowly drifts back producing an error, i.e., an overshooting glissade. The opposite can happen when the pulse is too weak and the eye drifts during the last part of the movement toward the target producing an undershooting glissade (Bahill et al., 1978). In this model, glissades appeared when there were pulse-step mismatches (Bahill and Stark, 1975; Bahill et al., 1975a).

The pulse and step are generated by a group of nuclei in the brainstem that are collectively called the brainstem burst generator (BBG; for review see Sparks, 2002). The superior colliculus (SC) and the OMV are the main inputs to the BBG that are responsible for executing the saccade (Figure 2). The OMV influences eye movements through its Purkinje cell responses, which provide the sole output of the cerebellar cortex. Their activity correlates accurately with the eye velocity of the upcoming saccade and aligns with the saccade ending (Thier et al., 2002; Herzfeld et al., 2015). It is therefore hypothesized that the OMV is responsible for keeping the pulse of the motoneuron drive accurate (Optican and Robinson, 1980). The OMV’s Purkinje cells tonically inhibit a part of the cerebellar fastigial nucleus called the fastigial oculomotor region (FOR). Activity in the contraversive FOR precedes the saccade, providing additional drive to the BBG and thus accelerating the eye movement. Later on, at the end of the movement, the ipsiversive FOR becomes active, choking off the drive to the ipsiversive BBG and thus stopping the eye movement (Noda et al., 1990; Fuchs et al., 1993; Voogd et al., 2012). When both FOR’s are lesioned, saccades become hypermetric in all directions, leading to the hypothesis that the OMV is responsible for ending the saccade (Robinson et al., 1993; Quaia et al., 1999) (for an extensive review on what stops the saccades see Optican and Pretegiani, 2017).

Notably, alternative models exist but they are mostly a variation on the pulse-step model of the saccadic control. For example a pulse-slide-step incorporates a force to the pulse that precedes and continues during the saccade, a force of the step that holds the eye at its new position and a force that counteracts the long time constants of the visco-elastic elements of muscles and the eye itself, i.e., the slide force (together the muscles and the eye are commonly referred to as “the plant”) (Collins, 1975; Miller and Robins, 1992). A full review of all models is out of the scope of this paper; for a comprehensive overview of the proposed models see Scudder et al. (2002).

Since the cerebellum seems to be responsible for timely termination of the pulse part of the drive of the saccade, we hypothesized that OMV lesions could lead to inaccuracies at the end of the saccade, i.e., the glissades. Here, we have studied the effects of the OMV lesions on glissade kinematics and on their rate of occurrence in primates. Furthermore, we have investigated to what extent glissades can be adapted, since the adaptation of eye movements based on errors from prior movements is an ability that critically depends on the cerebellum (Dash and Thier, 2014). We reasoned that during the glissade, a slower process of error feedback loop could be harnessed to adjust the step for endpoint correction. We hypothesized that if the contribution of the glissades were functional, it would be possible to observe a systematic contribution in the error distribution during an adaptation task.

## Materials and Methods

### Experimental Animals and Tasks in the Lesion Studies

For the lesion experiments, eye movements of four monkeys (Macaca mulatta, referred to as monkeys B, E, R, and S) were recorded using the scleral search coil tracking method (Judge et al., 1980) (spatial resolution < 0.1°, temporal resolution 1 ms). Animals were painlessly head restrained through an implanted head-post and trained to make visually guided saccades to targets (white dot with a diameter of 0.33° and a luminance of 12 cd/m^2^) while seated 22 cm from a computer monitor (21-in monitor; Flexscan F760i-W; frame rate: 72 Hz; 1280 × 1024 pixels). The eye was tracked unilaterally in all experiments. Data was collected from: both right and left eye but never simultaneously. Scleral search coils were calibrated by having the animal make visually guided eye movements to 9 points on the screen at different locations. The animal was visually monitored by an infrared camera inside the setup that allowed the experimenter to make sure that the animals were looking at the target. For details on surgical procedures and OMV lesions see Ignashchenkova et al. (2009).

Fixation targets were presented for 500 ms after which saccade targets appeared. The saccade targets had an eccentricity of 10° and were presented in one of eight different (0–315°) directions for a duration of 700 ms. Monkeys were trained to make saccades to the target for a fluid reward (water or juice, depending on the monkey’s preference) that they received if they moved their eyes to the target within 400 ms after its appearance.

Saccades were considered to be correctly executed when the animal fixated its gaze within a square region around the target of 2–2.5° side length. Animals were allowed to make a secondary saccade as long as fixation was reached inside the square window within 400 ms after the appearance of the target. Only primary saccades were considered for analysis. These values for the fixation window around the saccade targets were determined bearing in mind that our monkeys will undergo cerebellar lesions and their saccades will be very inaccurate after this procedure. Therefore, in the early days following the lesion the fixation size window was extended 2–4 fold.

### Experimental Animals and Tasks in the Saccadic Adaptation

For the saccadic adaptation experiment we used two different monkeys (Macaca mulatta, referred to as monkeys Mi and Mo). Animals were painlessly head restrained through an implanted titanium head-post and trained to make visually guided saccades to targets. The eye was tracked unilaterally in all experiments. Data was collected from the right eye in both monkeys.

The intra-saccadic step paradigm (McLaughlin, 1967) was applied for adaptive shortening or lengthening of saccades. Animals were trained to focus on a fixation dot in the center of the monitor (frame rate: 100 Hz, 1152 × 864 pixels), placed at the viewing distance of 52 cm. Eye movements were recorded in the dark with an infrared video eye tracker at 1000 Hz (Eyelink 1000 plus, SR Research). Standard Eyelink 5 point calibration with 10° eccentricity was used before every experiment. At the start of an adaptation trial, when fixation was detected, a saccade target appeared horizontal and ipsilateral from the fixation point in the periphery of the visual field at 10° eccentricity. The target was displaced inward or outward when the velocity of the eye exceeded 30°/s. Target displacements could have different amplitudes; classical inward or outward adaptation displacements were 2° (10° to 8° for inward adaptation; 10° to 12° for outward adaptation), whereas small saccadic adaptation displacements were 0.5° (10° to 9.5° for inward adaptation; 10° to 10.5° for outward adaptation). Each experiment contained between 550 and 600 trials. Saccades were considered to be correctly executed when the animal fixated its gaze within a circle region around the target of 3° diameter.

### Data Analysis

Eye movement data were analyzed using custom written MATLAB programs (MathWorks Inc.). Saccade onset and offset were detected on the basis of an adaptive velocity threshold, which consisted of 3 standard deviations (SD) of the noise during fixation. Position traces were differentiated with the ‘diff’ function of MATLAB (y = x_*i+*__1_−x_*i*_). Acceleration was acquired by further differentiating the velocity signal. We used a Savitzky-Golay filter for smoothing of the raw traces and a median filter for further smoothing of acceleration signals.

Only glissades immediately following the primary saccades were considered in this analysis. In accordance with previously published results, monkeys B, E, R, and S displayed some amount of corrective/secondary saccades, particularly in the early post-lesion condition. In the pre-lesion and late post-lesion condition, very few corrective saccades were detected, with the exception of monkey E who showed corrective saccades also in the baseline condition.

All glissades were selected by hand, based on the position and velocity profile of the eye movement by a single analyst. Selection of glissades was done without knowing which animal the file belonged to or whether it was from pre- or post-lesion. For the saccadic adaptation experiments, the trials were shuffled so the analyst did not know he was looking at saccades from the beginning of the adaptation or from the end. To be classified as a glissade, an overshoot or undershoot had to stand out clearly from the background noise as determined by the 3 SD baseline criterion; either in the form of a clearly distinguishable peak, directly attached to the saccade (Figure 1B), or as a shoulder visible as a deflection (Figure 1C) from the normal deceleration profile.

Post saccadic oscillations (PSO) are a phenomenon that can be present in the signal of video eye trackers. PSO’s are distinguishable from glissades because PSO’s show multiple peaks in the velocity profile whereas glissades show only one. Additionally, PSO’s are of higher velocity and amplitude than glissades (Kimmel et al., 2012). These features allow the analyst to mark glissades and ignore PSO’s. Glissades were classified as undershoots or overshoots relative to the post-saccadic fixation. If the early end of the saccade, i.e., the start of the glissade, passed the eventual fixation point, it was classified as an overshoot, whereas if after the early end of the saccade the eye moved further toward the fixation point, it was classified as an undershoot.

### Statistics

If statistical testing involved two groups, a student’s *t*-test was used unless the assumptions of normality and equality of variance were violated (Kolmogorov–Smirnov test or *F*-test for equal variances *p* < 0.05). In these cases, a Wilcoxon rank sum test was used. When more than two groups were involved, we used ANOVA with Bonferroni correction, unless any of the previously mentioned assumptions were violated; in which case we used the Friedman test. In cases of fractions, error bars represent Jeffreys interval and the *z*-test for proportions was used to determine significance. For circular statistics we used the *circle stats* toolbox for MATLAB. Since there is no *post hoc* test available for circular ANOVA, we performed the test on all groups individually and Bonferroni corrected the *p*-value (alpha = alpha/number of hypotheses). All statistical analysis was done using MATLAB.

To estimate the recovery times of the effect of the lesion on saccade amplitudes, glissade amplitudes and fraction of saccades with a glissade, we calculated the half-life of an exponential fit to these parameters. The formula [f(x) = a^∗^exp(−x/b) + c] was used for the exponential fit. Subsequently, the half-life (t_1__/__2_) was calculated with [t_1__/__2_ = tau^∗^ln(2)], where tau is b in the exponential function. The early post-lesion time interval was defined as 3 ^∗^ t_1__/__2_ based on the exponential fit to the saccade amplitudes in monkeys R and S. In monkey E, we calculated the half-life from the glissade amplitudes, since there was no significant fit to saccade amplitudes. Monkey B did not have significant fits to either saccades or glissades. Therefore, we used an average half life of the fits to saccade amplitudes from monkeys R and S to define the early post-lesion interval.

Error bars or shadings signify one standard deviation unless otherwise specified. Box and whisker plots contain median with box edges indicating the 25th and 75th percentile and the whiskers extend to the most extreme data points not considered outliers. Outliers determined as values larger or smaller than median ± 2.7σ (standard value for whiskers in MATLAB).

To evaluate whether lesions affected glissade parameters (e.g., glissade amplitude) related to the eight cardinal saccade directions, we replotted the rose plots, shown in Figure 7, as histograms which resulted in two distributions. We then calculated the Pearson’s correlation coefficient between the independent variables of the distributions.

For saccadic adaptation experiments, a linear regression was fitted using all glissadic over- or undershoots (analyzed separately) to analyze the glissade amplitude during the adaptation. To calculate the fraction of saccades with a glissade we used the first 50 and last 50 trials and applied *z*-test for proportions test to obtain the statistics.

## Results

To study the role of the OMV in the formation and characteristics of glissades we used a dataset from a previously published OMV lesion-study of 4 rhesus macaques (B, E, R and S; Ignashchenkova et al., 2009) and 2 of our own rhesus macaques (Mi and Mo). The aim of the original lesion study was to investigate the role of the OMV in spatial attention shifts, visual motion perception and the detection of luminance changes. Eye movements were tracked pre- and post-lesion using the sclera implanted search coil technique but the glissades were never analyzed (Judge et al., 1980). We used this dataset and analyzed saccades together with the occurrence and kinematics of glissades pre-lesion, early and late post-lesion. Saccades were classified in three categories; saccades without a glissade (Figure 1A), saccades with a glissadic overshoot (Figure 1B), and saccades with a glissadic undershoot (Figure 1C). In the pre-lesion condition 59% of saccades did not have any form of glissade (Figure 1A), 35% of saccades of all animals combined showed glissadic overshoots (Figure 1B), and 6% of saccades showed glissadic undershoots (Figure 1C, for percentage of individual animals see Supplementary Table 1). Glissadic undershoots had an average amplitude of 0.14 ± 0.08° and a duration of 13 ± 7 ms. Glissadic overshoots had an average amplitude of 0.21 ± 0.13° (SD) and a duration of 11 ± 5 ms. Because glissadic undershoots were relatively rare (Supplementary Table 1), in order to reach a significant power for our statistical comparisons we used glissadic overshoots alone in the analysis of the glissade endpoints, and pre- and post-lesion glissade comparisons. We do however present the analysis of the glissade kinematics separately for under- and overshoots, since these were not as constrained by the small size of the early post-lesion group particularly.

Notably, in our analysis we focused only on glissades immediately following the primary saccades. In accordance with previously published results, monkeys B, E, R, and S used in the lesion studies displayed corrective/secondary saccades, particularly in the early post-lesion condition. In the pre-lesion and late post-lesion condition, very few corrective saccades were detected, with the exception of monkey E who showed corrective saccades also in the baseline condition. These secondary saccades were not inspected for the presence or absence of glissades and are not included in any of our analysis.

### Saccades With and Without a Glissade Before Lesions

**FIGURE 3:**
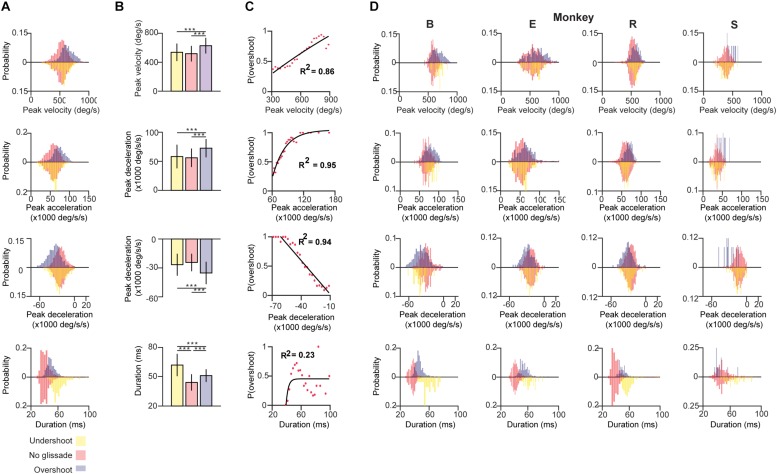
Kinematics of saccades with undershoot, overshoot or no glissade. **(A)** Probability density histograms of saccade kinematics of all animals combined. Red bars represent kinematic parameters of saccades without a glissade, purple bars represent saccades with glissadic overshoots and yellow bars saccades with glissadic undershoot. Red up and downward bars represent the same data to clearly compare the effect of undershoots and overshoots. **(B)** Average kinematics of saccades from all animals with glissade ± SD. **(C)** Linear or exponential regression analysis on the probability of having a saccadic with overshoots and kinematic parameters. **(D)** Histograms of the same kinematic parameters as represented in **(A)** for individual animals. “B,” “E,” “R,” and “S” denote each of the four monkeys. ‘^∗∗∗^’ symbols denote *p*-values of <0.001.

First, we compared the pre-lesion kinematic properties of saccades with and without glissadic over- and undershoots. Saccadic durations were measured as time from the onset of the saccade until complete fixation, including the glissade when present (blue arrow in Figures 1B,C). Figure 3A shows distributions of the kinematic parameters pooled across all monkeys for saccades without glissades, saccades with undershoots, and saccades with overshoots (depicted by negatively and positively deflected histograms, respectively). Individual animals are represented in Figure 3D in the same fashion.

Saccades with glissadic overshoots of all animals combined had higher peak velocities, accelerations, decelerations than saccades with glissadic undershoots and saccades without glissades (Figure 3B; all *p* < 0.001). Saccades with glissadic undershoots on the other hand were not different from saccades without glissades in the same categories (*p* > 0.01). Saccades without glissades had the shortest durations, followed by saccades with glissadic overshoots. Lastly, saccades with glissadic undershoots had the longest durations (*p* < 0.001 for all groups). Additionally, we explored the relation between the probability of a saccade having a glissadic overshoot and the same kinematic parameters of the saccade as discussed in Figures 3A,B. After binning peak velocities and calculating probabilities of glissadic overshoots associated with each bin, a regression model was fitted (Figure 3C). High peak velocities showed a positive linear relationship with the probability of glissadic overshoots (*R*^2^ = 0.86). Peak accelerations on the other hand showed a positive exponential relationship with the probability of glissadic overshoots (*R*^2^ = 0.95). Peak decelerations also had a linear relationship with probability of glissadic overshoot (*R*^2^ = 0.94). Lastly, glissadic durations did not show a clear relationship with probability of glissadic overshoots. The regression analysis was only performed for glissadic overshoot, because glissadic undershoots suffered from sample size constraints (Supplementary Table 1).

**FIGURE 4:**
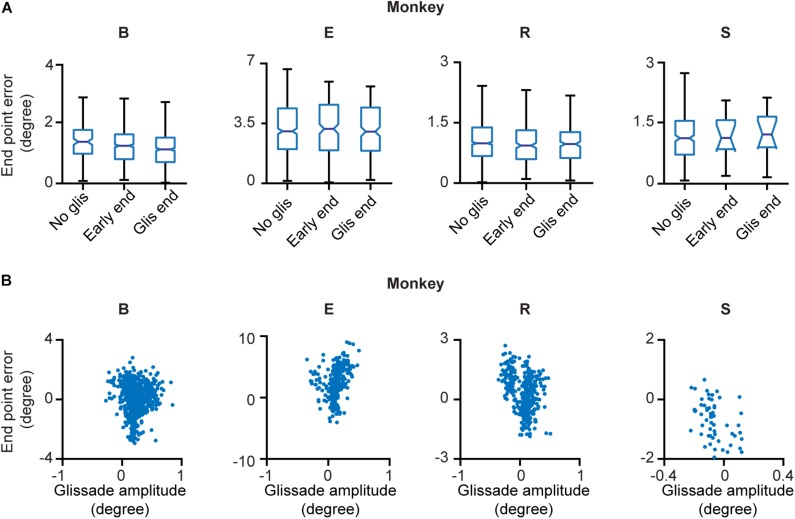
End point errors at onset and offset of the glissade and of saccades without a glissade. **(A)** The first box in every panel represents the average endpoint error of saccades without a glissade (“No glis”). The second box represents the end point error of the saccade at the onset of the overshooting glissade (“Early end”; green arrow in Figure 1B). The third box shows the end point error of the saccade at the end of the overshooting glissade (“Glis end”; blue arrow in Figure 1B). No significant differences were observed. **(B)** Scatter plots of signed end point saccade errors (negative – hypometric saccades; positive – hypermetric saccades) and glissade amplitudes (negative – undershoots; positive – overshoots) of individual animals. See Supplementary Table 2 for *p*-values of **(A)** and correlation coefficients of **(B)**. Labels “B,” “E,” “R,” and “S” denote each of the four monkeys.

Next, we explored whether glissades decrease endpoint errors. Endpoint errors were defined as the absolute position difference between the eye and the target: $\sqrt{}\,{\left(\mathrm{𝑋𝑒𝑛𝑑𝑝𝑜𝑖𝑛𝑡}-\mathrm{𝑋𝑡𝑎𝑟𝑔𝑒𝑡}\right)}^{2}+{\left(\mathrm{𝑌𝑒𝑛𝑑𝑝𝑜𝑖𝑛𝑡}-\mathrm{𝑌𝑡𝑎𝑟𝑔𝑒𝑡}\right)}^{2}$. We compared the endpoint errors at the beginning and end of overshooting glissades. No statistical difference was observed. There were also no differences when comparing endpoint errors of saccades with glissades where no glissade was present (Figure 4A and Supplementary Table 2 for *p*-values of the statistical tests). The end points of glissades and saccades were observed all around the target. Therefore, by chance, a hypermetric saccade with an overshooting glissade would somewhat decrease the end point error through the glissade. On the other hand a hypometric saccade with an overshooting glissade would increase the end point error. For this reason, we computed the coefficient of correlation between signed saccade endpoint (negative values represent hypometric saccades; positive values represent hypermetric saccades) and signed glissade amplitudes (negative values represent undershoots; positive values represent overshoots) (Figure 4B). We found no consistent relationship between end point error and glissade amplitude (see Supplementary Table 2 for correlation coefficient per each animal). This indicates that glissades do not provide additional accuracy by bringing the eye position closer to the desired endpoint.

### Recovery Time From OMV Lesion for Overshooting Glissades

Subsequently, we explored how both saccades and overshooting glissades change after lesions to the OMV. The extent of the lesions were distinct for individual monkeys, ranging from only partial ablation of lobules V-VIII of the OMV in monkey R to complete removal of lobules VI-VIII and partial removal of lob V and the fastigial nucleus in monkey B (for an overview of the extent of the lesion per monkey see Table 1). Lesions caused a clear but transient effect on saccadic amplitudes.

**TABLE 1:** Extent of lesion for individual animals.

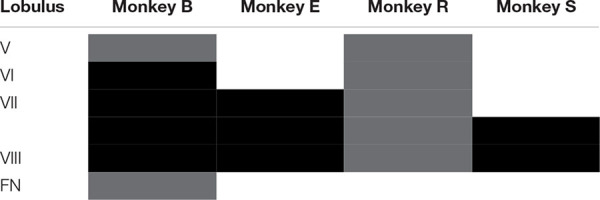

*Gray boxes represent partial ablation of the lobule and black boxes represent complete ablation of the lobule. For additional details, see Ignashchenkova et al. (2009).*

**FIGURE 5:**
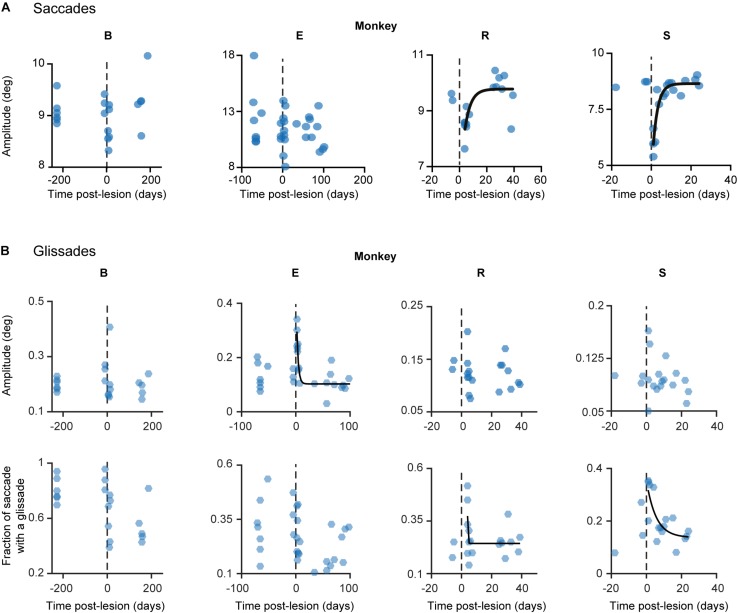
Development of lesion phenotypes over time. **(A)** Saccade amplitude per monkey plotted over time. Blue dots represent average saccade amplitude per experimental day including all directions pooled together, black fits represents exponential fit (f(x) = a^∗^exp(−x/b) + c). **(B)** Same as in **(A)** but the hexagons indicate glissade amplitude and fraction. See Supplementary Table 3 for *p*-values and coefficient of determination. Fraction is defined as the number of saccades containing a glissadic overshoot. Only overshooting glissades were used for this analysis. Labels “B,” “E,” “R,” and “S” denote each of the four monkeys.

Recovery of saccade amplitudes progressed exponentially in two out of four animals (Figure 5, black lines show a significant exponential [(f(x) = a^∗^exp(−x/b) + c; where t_1__/__2_ = tau^∗^ln(2), where tau = b in the previous formula] Supplementary Table 3 shows *R*^2^ and t_1__/__2_ for all animals). Exponential fits for saccades had a average half-life of 2.35 days ± 0.9 days (1 SD, unless otherwise specified), including only animals with a significant exponential fit (*n* = 2; R, S). Monkey E showed a clear exponential decay in glissade amplitude. Two out of four monkeys (R, S) showed a significant exponential decay of fractions of saccades with a glissade. For monkey B, who did not have any significant fits, we used the average half-life of saccades amplitude of monkeys R and S (for all *R*^2^, *p*-values and half-life parameters see Supplementary Table 3).

### Lesions of OMV Result in Mixed Phenotypes

**FIGURE 6:**
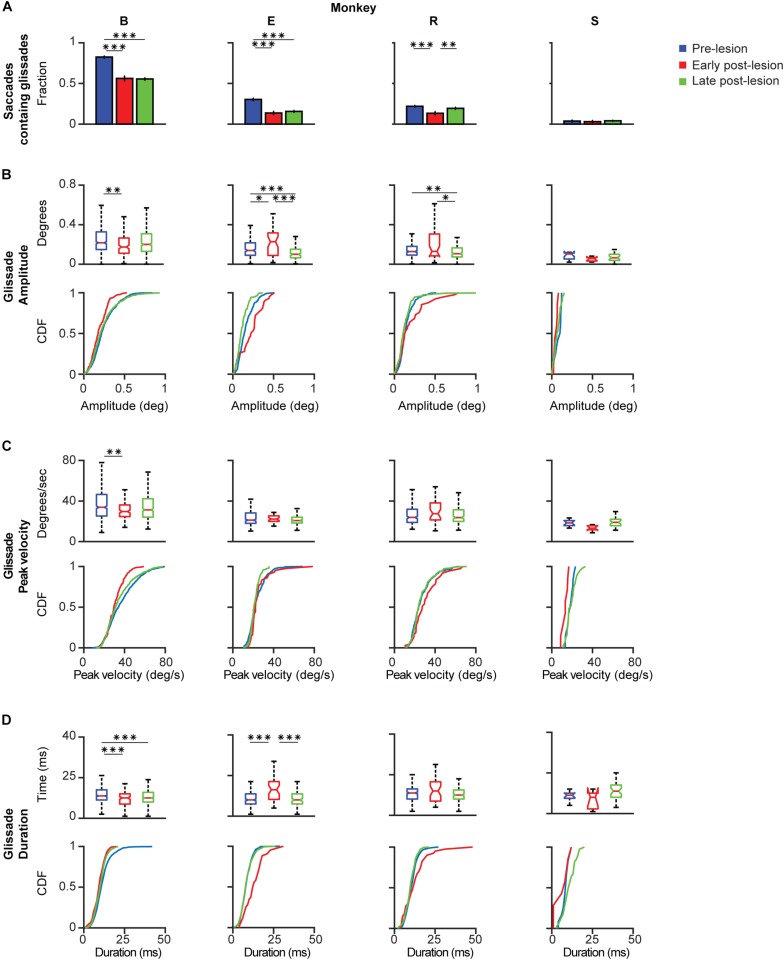
Post-lesion changes to glissadic overshoots. Glissade kinematics were sorted in a pre-, early, and late post-lesion groups. Early post-lesions was defined as the day of the lesion + 3 half-lives of the exponential fit in Figure 5, everything thereafter was classified as late post-lesion (for more details see section Results). Number of days that were classified as early post-lesion period is shown in the bottom row of Supplementary Table 3. **(A)** Fractions of saccades with a glissade for individual monkeys. Error bars represent Jeffrey’s interval for proportions. **(B)** Box plots and [Cumulative Distribution Function (CDF)] of glissade amplitudes for individual monkeys. Boxes edges indicate 25th and 75th percentile, box midline indicate the median. Whiskers extend to the most extreme data points not considered outliers. **(C)** Box plots and CDF of glissade velocities. **(D)** Box plots and CDF of glissade durations. Only overshooting glissades were used for this analysis. See Supplementary Table 4 for *p*-values. Labels “B,” “E,” “R,” and “S” denote each of the four monkeys. ‘^∗^’ symbols denote *p*-values of <0.05; ‘^∗∗^’ symbols denote *p*-values of <0.01; ‘^∗∗∗^’ symbols denote *p*-values of <0.001.

Based on the time course of the recovery period described above, we grouped the data in three time-epochs: pre-lesion, early post-lesion, and late post-lesion. The early post-lesion group was defined as lesion date +3 times the half-life of exponential fit for saccades; the period after that was categorized as late post-lesion (monkeys R and S). Monkey E did not show exponential recovery of saccade deficits. Therefore we used the fit for glissade amplitude to determine the early and late post-lesion epochs. Monkey B did not have any significant fits, therefore we used the average half-life of saccades of monkeys R and S. Since the effect of the lesion varied substantially between monkeys, only significant results are described for each animal individually (Figure 6). Only effects on glissadic overshoots are reported due to the earlier discussed constraints on glissadic undershoots.

Monkey B initially showed a very high fraction of saccades with glissades which decreased after the lesion and remained at this level in the late post-lesion period. Glissade amplitude initially decreased, but recovered in the late post-lesion period. It is notable that monkey B had a higher baseline fraction of glissades than the other animals. This could potentially be caused by repeated electrophysiological recordings of the SC that had been performed in this monkey prior to the current study (Ignashchenkova et al., 2004, see sections Materials and Methods and Discussion for details). However, it could also reflect the individual variability in glissades between animals. The glissadic peak velocity and duration of monkey B decreased early post-lesion. Glissadic durations showed a persistent decrease in the late post-lesion epoch, whereas glissadic peak velocity recovered to pre-lesion period values (for *p*-values see Supplementary Table 4).

Monkey E showed a decreased fraction of saccades with a glissade which stayed lowered in the late post-lesion period. Glissade amplitude decreased from early to late post-lesion period. Peak velocity is unaffected by the lesion. Glissade duration increased in the early post-lesion period and recovered to below their original values late post-lesion.

Monkey R’s fraction of saccades with a glissade decreased early post-lesion which recovered to pre-lesion values in the late post-lesion period. Average amplitude showed a stark increase in variability initially after the lesion. The glissade amplitude is decreased late post-lesion relative to early post-lesion. There was no effect on glissade peak velocity and duration.

Monkey S showed no change in glissade kinematics in any of the categories studied. This could be explained by the low percentage of glissadic overshoots that this animal made during all epochs of this study.

In summary, due to the large variability in the extent of the OMV lesions, the post-lesion changes to the kinematic parameters of glissades were highly heterogeneous. However, one common feature observed across several monkeys was that these effects regularly underwent a quick recovery to pre-lesion or close to pre-lesion values.

### Direction Specificity of Glissadic Overshoot Amplitude and Frequency Sorted for Saccade Directions

**FIGURE 7:**
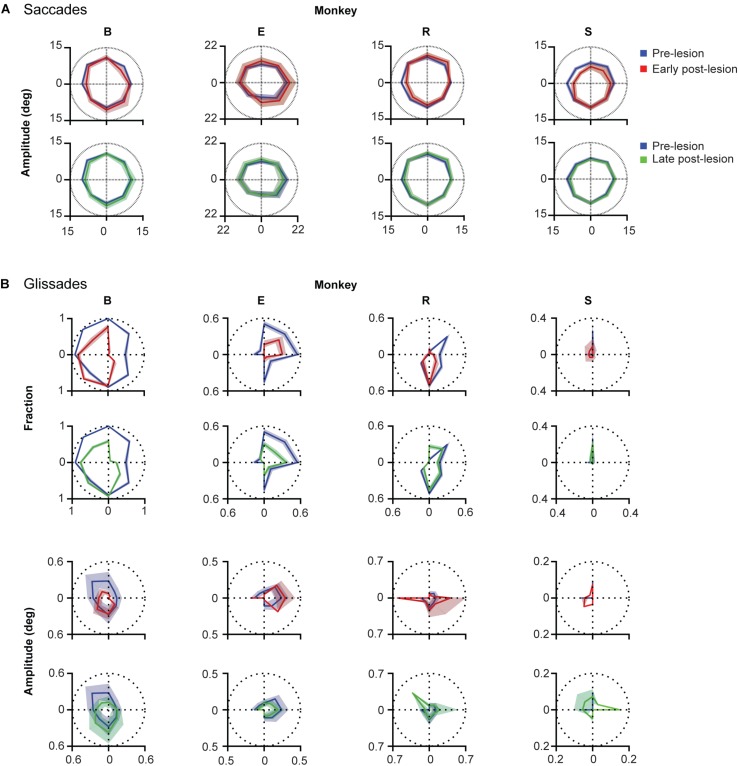
Direction specificity of lesion phenotype for glissades and saccades sorted for saccade direction. **(A)** Effect of lesion on saccade amplitude in different directions. Blue lines represent pre-lesion condition, red lines represent early post-lesion condition and green lines represent late post-lesion condition. **(B)** *Top two rows* show fractions of saccade with a glissade sorted for saccades in each of the eight cardinal directions. Shading of fractions represent Jeffrey’s interval. *Bottom two rows* show average glissade amplitude for saccades in each of the eight cardinal directions. Shading of amplitudes plots represent one standard deviation. Only overshooting glissades were used for this analysis. See Supplementary Table 5 for *p*-values and correlation coefficients between lesion time epochs. Labels “B,” “E,” “R,” and “S” denote each of the four monkeys.

Lesions to the OMV showed unique direction-dependent effects on saccade amplitudes (Figure 7A). Monkey B primarily showed hypometria in the upper half of the visual field. Monkeys E showed hypermetria in the right quadrants. Monkey R showed hypometria in the bottom left quadrant and hypermetria in the top right quadrant. Finally, monkey S showed hypometria in the top quadrants (Figure 7A).

To investigate whether the changes in glissade amplitude and fraction occured in the same direction after the lesion, we calculated the fraction of saccades with a glissade and the average glissade amplitude per 8 saccade directions (Figure 7B). This was done for all the lesion time epochs. To compare the directionality between time epochs, we calculated Pearson correlation coefficients of the directional distributions. First, we computed the correlation coefficient between the distribution of glissade amplitudes of the pre-lesion condition and the distribution of glissade amplitudes of the early post-lesion condition. If the correlation was significant we concluded that glissade amplitudes in the different time epochs had a preference for the *same saccade directions*. If the correlation was non-significant we concluded that the glissade amplitudes in the different time epochs had a preference for the *different saccade directions.* The same procedure was repeated for the other combinations of lesion time epochs: early-late and pre-late. The same analysis was also applied to the glissade numbers (fractions of saccade containing a glissade). We considered *p* = 0.02 as significant to correct for multiple testing, since we calculated correlations between the three groups (pre-early, pre-late, and early-late).

Monkey B showed no significant correlation in glissade amplitude and fraction between the pre- and early post-lesion period, but strong correlations in amplitude and fraction of glissade between early and late post-lesion (*R* = 0.96, 0.94; *p* < 0.001). This implies that the direction of amplitude and fraction of glissades changes from pre- to early post-lesion, but stays the same thereafter from early to late post-lesion (Figure 7B and Supplementary Table 5).

In monkey E, there was no correlation between amplitudes in any of the groups, showing glissade amplitudes changed in directional preference between all lesion epochs. A significant correlation between the fraction of glissades in the pre, early, and late post-lesion existed, implying that the directional distribution of the glissade fraction was similar in all lesion epochs (*R* = 0.83; 0.95; 0.82, *p* < 0.02 for all).

Monkey R showed no significant correlation in glissade amplitude between any of the groups thus implying that glissade amplitude in the different saccade directions changed between every lesion time epoch. Glissade fraction on the other hand was significantly correlated between the pre- and late post-lesion epochs (*R* = 0.86, *p* = 0.007) showing that the directionality of the fraction of glissades changed in the early post-lesion period and later recovered back to similar directionality in the late post-lesion period.

Monkey S showed no significant correlation in glissade amplitude between any of the groups, thus implying that glissade amplitude in the different saccade directions changed between every lesion time epoch. Glissade fraction, on the other hand, was significantly correlated between the pre- and late post-lesion epochs (*R* = 0.98, *p* < 0.001) showing that the directionality of the fraction of glissades changed in the early post-lesion period and later recovered back to similar directionality in the late post-lesion period.

**FIGURE 8:**
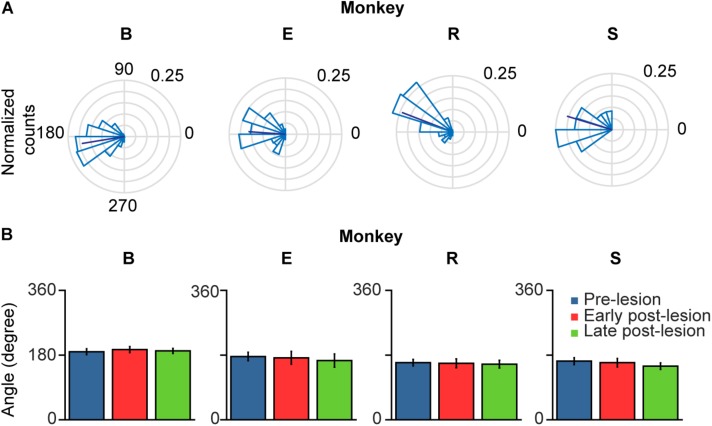
Polar plots of glissade angle relative to saccade. **(A)** Normalized polar histograms of glissade direction relative to saccade direction (all saccade directions set to 0°, glissade direction relative to saccade direction is defined as *θ_saccade_* – *θ_glissade_*) in the pre-lesion period. Dark blue line represents average vector of all glissade angles. **(B)** Mean glissade direction relative to saccade for pre-lesion and early and late post-lesion period. Only overshooting glissades were used for this analysis. Error bars represent one standard deviation. *P*-values of individual tests are in Supplementary Table 6. Labels “B,” “E,” “R,” and “S” denote each of the four monkeys.

When comparing glissadic overshoot direction with saccade direction it can be observed that glissades in all monkeys were in the opposite direction to the saccades that they were attached to (Figure 8A). Relative glissade directions did not change after lesioning of the OMV in any of the monkeys. To test the difference in angles between pre-, early, and late post-lesion epochs, the circular analog to the Kruskal–Wallis test with Bonferroni correction was used (Fisher, 1995). Figure 8B shows circular means and circular standard deviations of relative glissade angles. The lesion had no effect on the direction of glissades in any of the experimental epochs (see Supplementary Table 6 for *p*-values). These results show that glissade direction is strongly coupled to saccade direction, suggesting that glissades originate from the same motoneuronal drive signal as saccades.

### Glissade Characteristics During Saccadic Adaptation

**FIGURE 9:**
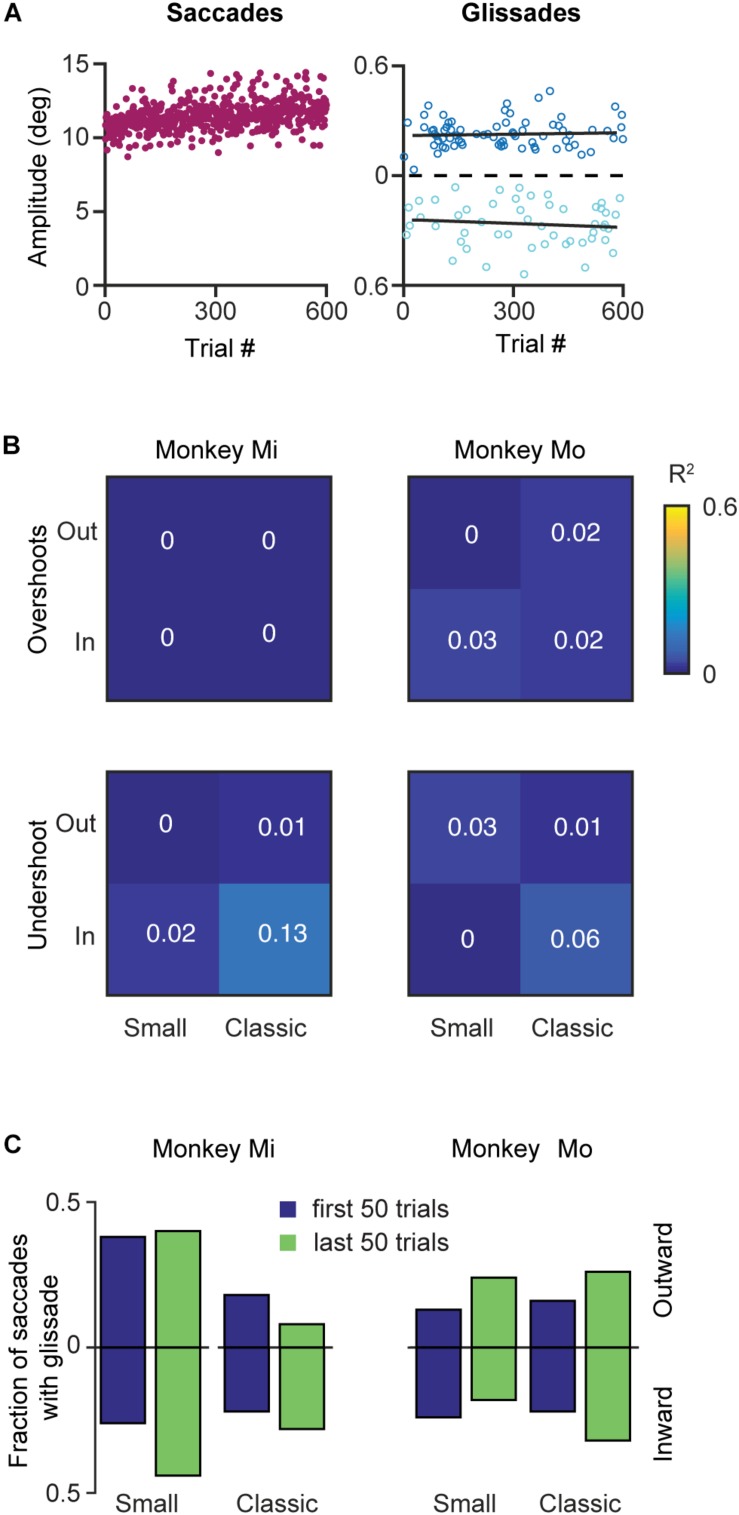
Effects of saccadic adaptation on glissades. **(A)** Example of a classical outward saccadic adaptation experiment from monkey Mo. *Left panel*, purple dots correspond to the saccade amplitudes gradually increasing over time. *Right panel*, dark blue dots display amplitude of the glissadic overshoots and light blue dots display amplitudes of the glissadic undershoots. Black lines represent a linear fit. **(B)** Top panels display *R*^2^ of linear fits of glissadic overshoot amplitudes during inward and outward saccadic adaptation with a classical and small adaptation step. The bottom panels display the same for undershoots. Monkey Mi did not make any glissadic overshoots. Labels “Mi” and “Mo” denote each of the two monkeys. **(C)** Fraction of saccades with overshooting and undershooting glissades pooled in the first and last 50 trials of the adaptation. Upward bars represent outward saccadic adaptation experiment and downward bars represent inward saccadic adaptation experiments for *p*-values see Supplementary Table 7.

Like many other movements, eye movements can be adapted by incorporating the errors from prior movements into the motor planning. This motor adaptation critically depends on the cerebellum (Optican and Robinson, 1980; Tseng et al., 2007). Therefore, we set out to investigate whether glissades are adaptable by using a classical saccadic adaptation paradigm and one where the displacement was in the same amplitude range as a glissade. To that end we have used two monkeys with an intact OMV (Mi, Mo), which we subjected to several saccade adaptation experiments. In these experiments eye position was tracked in the dark with infrared video eye tracker (Eyelink 1000 plus, SR Research). Video eye tracking can result in an overestimation of the glissades, because of the pupil wobble in the iris, which is visible as post-saccadic oscillations(PSO’s). Fortunately some discriminating features exist between pupil wobble and glissades. First, PSO’s produces several peaks in the velocity profile, and second, the amplitude of pupil wobble is generally larger than that of glissades (Kimmel et al., 2012). Figure 9A shows the results of an outward saccadic adaptation experiment where the target jumped from 10 to 12°. We applied four experimental protocols (see section Materials and Methods for details) performing both inward and outward adaptation experiments with a standard size adaptation step (2°, on a 10° primary saccade; where the amplitude of saccades gradually increased over time until it converged at 12° or decreased to reach 8°) and with a small adaptation step (0.5° on a 10° saccade; where the amplitude of saccades gradually increased over time until it converged at 10.5° or decreased to reach 9.5°) (McLaughlin, 1967; Pélisson et al., 2010). Although the saccade amplitudes robustly changed, glissade amplitudes from these saccades remained unaffected (Figure 9A, right panel, black lines depict linear regression). Since these monkeys made comparable numbers of undershoots and overshoots, we analyzed them separately in the regression analysis. Neither of the monkeys showed any change in glissadic overshoot or undershoot amplitude over the course of the adaptation experiments in any of the adaptation protocols (Figure 9B shows a matrix with the *R*^2^ values of all adaptation experiments).

To investigate if saccadic adaptation had any effect on the frequency of occurrence of glissades we compared the fraction of saccades with glissades at the beginning of the training to the fraction of saccades with glissades at the end of the adaptation (Figure 9C). We pooled overshoots and undershoots when studying the fraction of saccades with glissades because we constrained the analysis to only the first and last 50 trials when most of the gain change in saccadic adaptation is noticeable. None of the experiments had an effect on the fraction of saccades with a glissade (Figure 9C and Supplementary Table 7 for *R*^2^, *p*-values from *z*-test, and N numbers).

## Discussion

The cerebellum monitors the accuracy of eye movements and adjusts to errors in the gaze through saccadic adaptation (Optican and Robinson, 1980; Shadmehr et al., 2010). We investigated whether glissades, the occasional overshoots and undershoots that accompany a saccade, are actively controlled by the cerebellum, and to what extent they are susceptible to sensorimotor adaptation. We found mixed evidence for cerebellar involvement in glissade control. On the one hand we found that cerebellar lesions did impact the characteristics of the glissades. However, these features could not be influenced by saccadic adaptation, even though this form of motor learning has been shown to prominently depend on an intact cerebellum (Takagi et al., 1998; Barash et al., 1999).

To find out whether the cerebellum influences the kinematics of glissades we used an eye movement dataset from a cerebellar lesion study performed on four monkeys, and new data of two monkeys with an intact cerebellum. Analyzing the pre-lesion eye movements, we established that saccades with a glissade had higher peak velocities, accelerations, decelerations and longer durations than saccades without a glissade (Figure 3). Even though saccades with glissades took more time to reach fixation, the glissade itself did not contribute to bring the eye closer to the target (Figure 4). These characteristics were consistent across all monkeys, indicating that glissades do not improve gaze accuracy. Furthermore, glissade direction was heavily dependent on saccade direction. Due to a substantial variation in lesion severity (Table 1) we identified a diverse phenotype of changes in the glissade profiles. Depending on the animal, changes in frequency of glissade occurrence, amplitude of glissades, peak velocity and duration could be observed (Figure 6). Recovery of glissade abnormalities followed clear exponential progression in monkeys E, R, and S. On average the acute recovery time was estimated at ∼7 days post-lesion for those two monkeys. With the exception of monkey B, glissades were most pronounced in the early post-lesion period. Monkey B displayed an unusually high frequency of glissades in the pre-lesion condition. This monkey underwent repeated electrophysiological recordings from the SC. It is known that, besides the brainstem, the SC also provides mossy fiber input to the OMV and that electrical stimulation of the SC is sufficient to elicit saccades (Sparks and Hartwich-Young, 1989). Further, it has been observed that muscimol injections in the intermediate layers of the SC can alter saccade trajectories (Aizawa and Wurtz, 1998). Considering this, we suspect that the high baseline frequency of glissades was a result of the incremental damage from SC experiments. Surprisingly, this initial high frequency of glissades was decreased post-lesion; we speculate that the cerebellum had initially overcompensated for the output of the damaged SC, which was diminished when the OMV was also lesioned. Similar phenotypes have been observed in other studies investigating double lesions. It has been shown that cats with an ablated paravermis initially display tonic flexion of the ipsilateral limb, high stepping and ankle instability when walking, but recover from most of these deficits within 2–5 days. However, animals with pre-existing lesions to the red nucleus, which receives inputs from the cerebellum, showed no tonic flexion symptoms (Yul, 1972). Another example exists in the visual system, where unilateral cortical ablation, resulting in contralateral hemianopia (blindness to one half of the visual field), can be improved by lesioning the SC contralateral to the cortical lesion; this has been reported as the Sprague effect (Sprague, 1966). Notably, this explanation for the higher baseline rate of the glissades is merely a hypothesis and further experiments with specific SC lesions are needed to establish a causal relationship. Although unlikely, we cannot exclude that this high fraction of glissades is merely a reflection of individual variability in monkeys. Interestingly, the recovery times of the changes to the glissades which we observed in our study were similar to the ones reported for the paravermal lesions discussed above. As seen in the results, the post-lesion increase in the fraction of saccades with a glissade was transient and recovered with a time course similar to that of the saccade recovery-time. The latter was in line with previous studies, which show that saccadic dysmetria is reversed quickly following lesions to the OMV (Takagi et al., 1998; Barash et al., 1999; Ignashchenkova et al., 2009). The compensatory mechanism enabling the recovery is currently unknown, but two studies of natural lesions to the FOR in humans (e.g., through hemorrhage or tumor) do not show any recovery in saccadic dysmetria over time (Büttner et al., 1994; Straube et al., 1995). The FOR receives direct mossy fiber inputs from the same areas in the nucleus reticularis tegmenti pontis and paramedian pontine nucleus, which provide mossy fiber inputs to the OMV. Strikingly the mossy fiber projections to the OMV and FOR also share similar topographical organization (Yamada and Noda, 1987; Noda et al., 1990). Cells from the saccade region in the NTRP discharge 20–30 ms before the onset of a saccade in a direction-selective manner (Crandall and Keller, 1985). Additionally, the FOR receives collaterals from the part of the medial accessory olive that also provides climbing fibers toward the OMV (Ikeda et al., 1989; Noda et al., 1990). Therefore, these olivo-nuclear projections likely carry the same information as the OMV receives. Together these inputs could be utilized to compensate for the ablation of the OMV through plastic changes in the FOR.

To find out to what extent glissades are susceptible to sensorimotor adaptation we investigated whether glissades can be adapted using an inward and outward saccadic adaptation paradigm in two monkeys with intact OMV. We rationalized that if glissades reflect a mismatch between pulse and step, a neural correlate of the mismatch could conceivably *a posteriori* correct for saccadic vector imprecision. Given that inward and outward adaptation paradigms rely on different structures and affect saccade kinematics in a different way, we tested both conditions. Based on Shadmehr’s (Ethier et al., 2008) adaptation data, which suggest that inward adaptation affects saccade kinematics by decreasing the height of the pulse, we expected to see a change in the glissades in this test. However, we found that glissades were not susceptible to adaptation through either a standard saccadic adaptation paradigm (10% of primary saccade gain) or one where the adaptation step is in the same order of magnitude as the glissade itself (0.5°). Furthermore, the frequency of the glissades also remained unchanged in both outward and inward adaptation.

From this, we conclude that glissades are not readily adaptable, at least using the classical saccadic adaptation paradigm. It remains an open question whether exponential target drift induced immediately at saccade offset (similar to visual perturbations used to elicit ocular following responses) could lead to glissade adaptation. Another manipulation that could affect the glissade kinematics and probability is a change in the spatiotemporal contrast, which we know can be increased at low temporal frequencies (Westheimer and McKee, 1975). We hope to see future experiments addressing both of these questions.

The lesion dataset and the adaptation dataset were obtained using different eye tracking methods. The former with the sclera-embedded search coil technique and the latter using the video eye-tracking method. The method of eye tracking is of major importance when measuring glissades, since these movements are close to spatial and temporal resolution limits of most tracking methods. Advantages and disadvantages of different methods for precise eye tracking have been a topic of a discussion for some time (van der Geest and Frens, 2002; Kimmel et al., 2012) and some inconsistencies have been observed when measuring glissades with different eye tracking methods. Kimmel et al. (2012) compared the sclera-embedded search coil technique with the video eye tracking based on pupil center of mass by simultaneously recording from the same eye in macaque monkeys. They showed that pupil based methods are more sensitive to recording PSO’s. When the eye stops, the pupil occasionally wobbles in the iris at the end of the eye movement resulting in a PSO. Therefore, pupil based eye trackers for saccades where pupil wobble is present display a signal which is a combination of actual eye movement and pupil wobble. These PSO’s could easily be mistaken for glissades, although no actual movement of the eye takes place during this type of oscillation (Kimmel et al., 2012; Nyström et al., 2013). Based on these studies, the sclera-embedded search coil technique seems to be the preferred method for measuring glissades, since it does not suffer from errors resulting from wobble of the pupil inside the iris. In order to reduce errors resulting from our eye tracking approach we employed the following steps: (1) Glissades were distinguished from PSO’s (and confirmed by an additional experimenter via visual inspection; see section Materials and Methods for details) in that they showed a single rather than multiple peak(s) in their velocity profile and that their amplitude and velocity were lower than those of PSO’s (Kimmel et al., 2012); (2) Saccades within each experiment were shuffled so that the analysis was blinded to the trial number. This way the glissades were labeled without *a priori* knowledge of whether they belonged to saccades recorded at the beginning or at the end of the experiment. Taken together, the distinct velocity profiles allowed us to mark glissades alone and ignore PSO’s, and trial shuffling ensured impartial analysis. Therefore, we are confident that the observed lack of changes in the glissade amplitude and fraction, in any conditions throughout the adaptation, was not due to any systematic mislabeling of the PSO’s.

It is possible that other cerebellar or non-cerebellar regions play a role in glissade formation. A systems model by Bahill et al. (1978) suggests that glissades are a product of errors in the width of the pulse (i.e., duration) in the pulse-step control signal of saccades. In Bahill’s model, the pulse brings the eye quickly to the new target and the step keeps the gaze fixed in the new position. The production of a consistently accurate pulse is dependent on the OMV (Optican and Robinson, 1980), whereas the step component is dependent on the flocculus, nucleus prepositus hypoglossi and medial vestibular nuclei (Sparks, 2002). Indeed, lesion studies of the flocculus cause inability to keep the gaze in an eccentric position (Zee et al., 1981). The gaze drift after a saccade in floculecotomied monkeys has a duration of 40–150 ms, whereas glissades are in the range of 10–40 ms. Furthermore, post-saccadic ocular following of a persistent full-motion stimulus is dependent on the flocculus (Optican and Miles, 1985; Optican et al., 1986). Onset of following has an initial latency of 50–60 ms after the saccade, which is nullified after adaptation and is also abolished after flocculectomy. Glissades on the other hand are always appended directly to the saccade and their direction is strongly coupled to the direction of the saccade.

In Bahill’s framework, a mismatch between the amplitude of the pulse and the step explain the direction and amplitude of the glissades. Moreover, it is presumed that the step results from the neural integration of the pulse. Systematic pulse integration errors would lead to systematic changes in glissades, meaning glissades of similar saccades should have a similar direction (over- or undershooting). In our case, we have discovered that saccadic adaptation paradigms did not affect glissade systematically, meaning that whichever neural process matches the pulse and the step, an inherent source of variability results in a stable proportion of glissades, with kinematic parameters largely independent of target error of the initial saccade.

We argue that our data suggest that glissades are a product of an imprecision of the pulse integration, as the parameters of the glissade do not seem to be independently controlled. An undershooting glissade occurs when the pulse is insufficient to match the step and thus the eye slowly drifts to the step equilibrium position. Conversely, in overshooting saccades the pulse is in excess, adding an overshoot at the end of the saccade back to the encoded step amplitude. Presumably, the OMV keeps saccade amplitudes accurate by complementing the drive of the pulse width, hence the commonly observed hypometrias after lesions of the OMV. It has been hypothesized that the OMV achieves this by tracking the saccade as it progresses and choking off the drive and stopping the pulse when the target is reached (Optican and Pretegiani, 2017). Consequently, when the OMV is ablated, saccades become hypometric, as we argued, due to inaccurate termination of the pulse. The glissades kinematics, however, do not show such consistent change. In our experiments two out of four monkeys display more prominent glissades (monkey E and R). This change is far less dramatic than the saccade impairment. In the other two monkeys, glissade fraction is either stable or decreases (for a possible explanation of this phenomena in monkey B see the section Discussion above). That in itself suggests that the OMV does not play any functional role in determining glissade parameters. In order to fully investigate the role of the cerebellum in the glissades more studies are needed, specifically in the contributions of the flocculonodular lobe.

In summary, our results show that saccades with glissades had a longer duration, higher peak velocities, and faster peak decelerations and accelerations. Lesioning the OMV had an effect on glissade frequency, amplitude, peak velocities and duration. Furthermore, these effects recovered in an exponential fashion over the course of days. Glissade deficits, like saccades, were more pronounced in some directions. Lastly, glissades were not adaptable using either a classical inward or outward saccadic adaptation paradigm, nor in an adaptation paradigm with adaptation step commensurate with the glissade. Taken together, our findings indicate that glissades are the consequence of an error of the oculomotor system, rather than a functional movement controlled by the cerebellum. Specifically, it seems likely that glissades are a pulse integration error of the pulse-step command caused by inaccurate termination of the pulse.

## Data Availability

The datasets for this study will not be made publicly available because they are still in use for other research.

## Ethics Statement

All experimental and surgical procedures complied with the NIH Guide for Care and Use of Laboratory Animals (National Institutes of Health, Bethesda, MD, United States), and were approved by the institutional animal care and use committee of Tübingen (RP Tübingen, FG Tierschutz; lesion studies) and of the Royal Netherlands Academy of Arts and Sciences (saccadic adaptation studies).

## Author Contributions

NF and AI designed and performed the experiments. NF analyzed the data. AB, CDZ, and PT supervised the project. MN and CDZ suggested the hypothesis and provided conceptual feedback. NF, CDZ, and AB wrote the manuscript.

## Conflict of Interest Statement

The authors declare that the research was conducted in the absence of any commercial or financial relationships that could be construed as a potential conflict of interest.

## Footnotes

1 https://imotions.com/blog/top-eye-tracking-hardware-companies
