# Supplementary material for: Glissades Are Altered by Lesions to the Oculomotor Vermis but Not by Saccadic Adaptation
Source: Front Behav Neurosci. 2019 Aug 23;13:194. doi: 10.3389/fnbeh.2019.00194 (PMC6716469; doi:10.3389/fnbeh.2019.00194)
Supplement: Supplementary file 1 [file Data_Sheet_1.docx]

*Supplementary Data*

***Supplementary Table 1:*** Percentage of saccades with glissade undershoots or overshoots in the pre-lesion condition.

|  | *Boris* |  | *Elvis* |  | *Rocky* |  | *Spock* |  |
| --- | --- | --- | --- | --- | --- | --- | --- | --- |
|  | *N* |  | *N* |  | *N* |  | *N* |  |
| *total* | 882 | 100% | 874 | 100% | 1473 | 100% | 364 | 100% |
| *overshoots* | 704 | 80% | 256 | 29% | 300 | 20% | 12 | 3% |
| *undershoots* | 29 | 3% | 33 | 4% | 108 | 7% | 46 | 13% |
| *no glis* | 149 | 17% | 306 | 35% | 1065 | 72% | 306 | 84% |

***Supplementary Table 2****: p-values of ANOVA and correlation coefficients shown in* ***Figure 4****.*

| *ANOVA* | *No glis - Early end* | *Early end - Glis end* | *No glis - Glis end* | *Correlation*  *coefficient* |
| --- | --- | --- | --- | --- |
| *B* | 1 | 0.30 | 0.37 | -0.008 |
| *E* | 0.52 | 1 | 0.96 | 0.32 |
| *R* | 0.07 | 0.44 | 1 | -0.38 |
| *S* | 1 | 1 | 1 | -0.42 |

***Supplementary Table 3****: Decay time constants, coefficients of determination and p-values from regression analysis on saccade amplitude, glissade amplitude and glissade fraction over time for exponential fits (black lines) in* ***Figure 5****. Decay time constants were calculated only for significant fits (significant p-values indicated in bold).*

|  | **B** | | | **E** | | | **R** | | | **S** | | |
| --- | --- | --- | --- | --- | --- | --- | --- | --- | --- | --- | --- | --- |
|  | **R^2^** | **t_1/2_** | **p** | **R^2^** | **t_1/2_** | **p** | **R^2^** | **t_1/2_** | **p** | **R^2^** | **t_1/2_** | **p** |
| Saccade amplitude | 0.34 | ~ | 0.19 | 0.07 | ~ | 0.54 | 0.62 | 3 | **0.001** | 0.85 | 1.7 | **>0.001** |
| Glissade amplitude | 0 | ~ | 0.94 | 0.81 | 2 | **>0.001** | 0.19 | ~ | 0.08 | 0.11 | ~ | 0.21 |
| Glissade fraction | 0.018 | ~ | 0.41 | 0.17 | ~ | 0.19 | 0.34 | 0.2 | **0.02** | 0.6 | 3.8 | **0.003** |
| Time early post-lesion | 7 days | | | 6 days | | | 9 days | | | 6 days | | |

***Supplementary Table 4****: Comparison of glissade kinematics between lesion groups. p-values of data reported in* ***Figure 6.*** *Fractions were tested with z-test test for proportions. Amplitude, velocity, and duration were compared by ANOVA’s with Bonferroni corrections as post-hoc test. N in top row represent number of glissades used per group (pre, early, late). Significant p-values indicated in bold.*

|  | **B (N=149)** | | | **E (N= 43)** | | |
| --- | --- | --- | --- | --- | --- | --- |
|  | Pre- early | Early- late | Pre- late | Pre- early | Early- late | Pre- late |
| Fractions | **<0.001** | 0.83 | **<0.001** | **<0.001** | 0.39 | **<0.001** |
| Amplitude | **0.002** | 0.07 | 0.34 | **0.045** | **<0.001** | **<0.001** |
| Velocity | **0.002** | 0.85 | 0.05 | 1 | 0.16 | 0.22 |
| Duration | **<0.001** | 0.59 | **<0.001** | **<0.001** | **<0.001** | 1 |
|  | **R (n=41)** | | | **S (n=20)** | | |
|  | Pre- early | Early- late | Pre- late | Pre- early | Early- late | Pre- late |
| Fractions | **<0.001** | **0.01** | 0.13 | 0.69 | 0.49 | 0.77 |
| Amplitude | 1 | **0.02** | **0.002** | 0.20 | 0.83 | 0.59 |
| Velocity | 0.15 | 0.19 | 1 | 0.06 | 0.005 | 1 |
| Duration | 0.49 | 0.65 | 1 | 1 | 0.10 | 0.14 |

***Supplementary Table 5****: Statistical analysis of the data presented in* ***Figure 7****. Fraction denotes the fraction of saccades with a glissade in respect to saccades in each of the eight cardinal directions. Glissade amplitude was calculated for glissade containing saccades in each of the eight cardinal directions. We replotted the rose plots as histograms which resulted in two distributions (for example, pre and early). From these we calculated the correlation coefficient between those two distributions. R-values represent Pearson correlation for the fraction and amplitude as defined above compared between different lesion time epochs (pre-lesion vs early post-lesion, i.e. Pre-Early; pre-lesion vs late post-lesion, i.e. Pre-Late; and early post-lesion vs late post-lesion, i.e. Early-Late) P-values represent significance of the correlation coefficient (significant p-values indicated in bold).*

|  | | **B** | | | **E** | | | **R** | | | **S** | | |
| --- | --- | --- | --- | --- | --- | --- | --- | --- | --- | --- | --- | --- | --- |
|  |  | Pre-  Early | Pre-  Late | Early-  Late | Pre-  Early | Pre-  Late | Early-  Late | Pre-  Early | Pre-  Late | Early-  Late | Pre-  Early | Pre-  Late | Early-  Late |
| Fraction | R | 0.55 | 0.44 | 0.94 | 0.83 | 0.95 | 0.82 | 0.69 | 0.86 | 0.73 | 0.77 | 0.98 | 0.80 |
|  | p | 0.16 | 0.28 | **<0.001** | **0.01** | **<0.001** | **0.01** | 0.06 | **0.007** | 0.04 | 0.02 | **<0.001** | 0.02 |
| Amplitude | R | 0.23 | 0.09 | 0.96 | 0.85 | 0.87 | 0.71 | 0.54 | 0.40 | 0.09 | 0.64 | 0.10 | -0.08 |
|  | p | 0.61 | 0.85 | **<0.001** | 0.03 | 0.02 | 0.11 | 0.22 | 0.37 | 0.85 | 0.09 | 0.81 | 0.86 |

***Supplementary Table 6****: Glissade direction relative to saccade direction compared between pre-lesion, early and late post-lesion. p-values of circular Kruskal-Wallis test of* ***Figure 8B****. Tests were considered significant when p<0.0167 (Bonferroni correction for 3 tests).*

|  | *pre-early* | *early-late* | *pre-late* |
| --- | --- | --- | --- |
| *B* | 0.94 | 0.41 | 0.28 |
| *E* | 0.42 | 0.38 | 0.52 |
| *R* | 0.85 | 0.84 | 1 |
| *S* | 0.51 | 0.68 | 0.21 |

***Supplementary Table 7****: Coefficient of determination (R^2^) for regression between glissade amplitude, p-values of z-test for proportions used to compare first and last 50 trials and the number of trials per saccadic adaptation experiment* ***Figure 9B****.*

| *Monkeys* | *Outward* | | *Inward* | |
| --- | --- | --- | --- | --- |
|  | *Classic* | *Small* | *Classic* | *Small* |
|  | ***P-value z-test*** | | | |
| *Mi* | 0.14 | 0.84 | 0.49 | 0.06 |
| *Mo* | 0.22 | 0.05 | 0.26 | 0.46 |
|  | ***# trials*** | | | |
| *Mi* | 600 | 565 | 600 | 600 |
| *Mo* | 600 | 600 | 600 | 600 |
